# Organizational Silence and Work Engagement Among Chinese Clinical Nurses: The Mediating Role of Career Calling

**DOI:** 10.1155/jonm/7522633

**Published:** 2025-06-13

**Authors:** Wenfen Zhu, Heping Liao, Qian Wu

**Affiliations:** ^1^Department of Primary Nursing, School of Nursing, Chongqing Medical University, Chongqing, China; ^2^Department of Nursing, The First Affiliated Hospital of Chongqing Medical University, Chongqing, China

**Keywords:** career calling, clinical nurses, organizational silence, work engagement

## Abstract

**Aim:** This study aimed to explore how organizational silence affected work engagement in Chinese nurses and examining the impact of career calling on mediating the relation.

**Background:** Work engagement of nurses is vital for the quality of healthcare service. Organizational silence has been suggested to be negative for work engagement, but it remains elusive if organizational silence may reduce career calling and work engagement among nurses.

**Method:** This study used the self-report questionnaire for data collection in Chongqing City, China. Structured questionnaires were adopted to measure clinical nurses' organizational silence, work engagement, and career calling. Our research hypotheses were verified through using the structural equation model.

**Result:** A total of 1858 valid questionnaires were collected. Organizational silence showed significant and negative relation with work engagement and career calling of clinical nurses (*p* < 0.01). Career calling played a role in mediating the relation of organizational silence with work engagement.

**Conclusion:** Reducing organizational silence is important for promoting the work engagement of clinical nurses, and career calling exerts a key effect on interpreting the mechanism of organizational silence in reducing nurses' work engagement.

**Implications for Nursing Management:** Hospital managers must focus on reducing nurses' organizational silence to increase clinical nurses' work engagement and career calling.

## 1. Introduction

Work engagement has been defined as a positive, fulfilling, and work-related state characterized by energy, dedication, and total commitment to work [1, 2]. Highly engaged employees typically demonstrate strong motivation, enthusiasm, and engagement at work, which is associated with better performance, better outcomes, and lower intention to leave [3, 4]. In clinical settings, nurses' work engagement not only has a direct impact on patient health outcomes [5] but also can improve job satisfaction, performance, and quality of care [6, 7], while reducing turnover intentions and work burnout [6, 8]. Therefore, exploring the factors affecting nurses' work engagement has important theoretical and practical value.

Among these factors, organizational silence has received increasing attention. Organizational silence is a collective-level behavioral phenomenon in which individuals consciously withhold opinions, suggestions, or concerns, especially when faced with organizational problems or challenges [9, 10]. It refers to the lack of expression and interaction when employees choose to remain silent and unwilling to contribute to the development of the organization in the face of major issues in the organization [10, 11], which can lead to insufficient communication, dissatisfaction, increased stress, and lack of work performance among employees, and it is a potential hindrance to positive employee behavior within the organization [12, 13]. Organizational silence and work engagement are important topics for organizations to achieve their desired goals [14]. In clinical nursing, organizational silence among nurses may manifest itself as a reluctance to report errors, make suggestions for improvement, etc., thus hindering communication, increasing stress, preventing meaningful dialog within the healthcare team, and ultimately affecting nurse engagement. Although studies have found that organizational silence is negatively correlated with employee work engagement [15, 16], there are still fewer relevant studies in the clinical nurse population, and the mechanism by which organizational silence reduces nurses' work engagement needs to be further investigated. Therefore, this study examined the relationship between organizational silence and work engagement among clinical nurses and explored the mediating role of career calling in the relationship.

The Job Demand-Resource (JD-R) model states that behavioral outcomes of work (e.g., work engagement) are the result of a dynamic interaction between job demands (e.g., emotional needs and communication suppression), job resources (e.g., performance feedback, interpersonal relationships, and autonomy), and personal resources (e.g., self-efficacy) [1, 17].

Organizational silence can be viewed as a job demand that depletes psychological energy by restricting employees' expression, inhibiting feedback, and diminishing employees' autonomy and meaning over the content of their work [12, 13]. Conversely, career calling has been defined as a highly internalized “personal resource,” which may be a motivator for work engagement [18].

Career calling has been defined as a belief that refers to the work value, representing the individual's passion and motivation for work, emphasizing that an individual's work will fulfill personal values and satisfy social needs [19, 20]. Nurses with a strong career calling typically exhibit higher intrinsic motivation and are more likely to experience job satisfaction and emotional fulfillment [21, 22]. Studies have documented that career calling not only positively predicts nurses' career satisfaction and job thriving [23, 24] but also positively correlates with work engagement [25, 26]. There is a negative correlation between organizational silence and career calling, and organizational silence reduces the level of employees' career calling [21], but there are still relatively few empirical studies related to this for clinical nurses [21, 27]. In addition, studies have shown that career calling is a key mediator between organizational environments and job performance [28, 29]. By reinforcing an individual's commitment to his or her profession, career calling can act as a psychological buffer against unfavorable workplace factors [30]. Thus, career calling may have an important mediating role in the relationship between organizational silence and nurses' work engagement among clinical nurses.

Based on the theoretical and empirical evidence presented above, this study proposed the following hypotheses: Hypothesis 1: organizational silence is significantly negatively correlated with nurses' level of work engagement, i.e., the higher the level of organizational silence of nurses, the lower their level of work engagement and Hypothesis 2: career calling mediates the relationship between organizational silence and work engagement. Specifically, the higher the level of organizational silence, the lower the nurses' sense of career calling, which in turn leads to a decrease in their level of work engagement. In conclusion, by introducing the psychological resource variable of career calling, this study attempted to reveal how organizational silence indirectly acts on work engagement by influencing individuals' intrinsic motivation and to provide theoretical support for enhancing the occupational motivation and organizational management effectiveness of nursing teams.

## 2. Methods

### 2.1. Subjects and Data Extraction

This study was a secondary analysis based on an anonymous clinical nurse satisfaction survey conducted as part of routine quality monitoring by the Nursing Departments of four tertiary hospitals in Chongqing, China. Data were collected electronically via a web-based questionnaire platform between May and August 2024. With the support of the hospitals' nursing managers, the survey was distributed to eligible nurses, and participation was entirely voluntary. All participants provided informed consent prior to completing the survey, and they were assured of confidentiality and anonymity. The average completion time was 5–8 min. Ethical approval for the secondary use of this anonymized dataset for research purposes was obtained from the Ethics Review Committee of the First Affiliated Hospital of Chongqing Medical University (Approval no. 2024-345-01). Responses were reviewed and invalid questionnaires were excluded. The criteria for invalid questionnaires were as follows: (1) submitted within 2 min or more than 15 min; (2) same answers or same pattern of answers for all items (e.g., repeated answers 1, 2, and 3); and (3) any missing items in the questionnaire.

### 2.2. Measures

#### 2.2.1. Organizational Silence

Organizational silence was measured by the Nurses' Organizational Silence Assessment Questionnaire developed by Chinese scholar Yang Jing [31], which contains four dimensions: negativity silence, defensiveness silence, prosociality silence, and indifference silence, with 4 entries in each dimension, resulting in a total of 20 entries. Organizational silence was scored on a Likert 5-point scale between 1 (never) and 5 (always), and a greater total score indicated more significant phenomenon of nurses' organizational silence. The scale has good reliability and validity in the nurse population [31], and the Cronbach's alpha coefficient of negativity silence, defensiveness silence, prosociality silence, indifference silence, and organizational silence in this study is 0.937, 0.954, 0.941, 0.900, and 0.914, respectively.

#### 2.2.2. Work Engagement

A 9-item Version of the Utrecht Work Engagement Scale (UWES) put forward by Schaufeli et al. in 2006 [32] was adopted for measuring work engagement. UWES can be adopted for measuring people's work engagement levels in different occupations. Fuye et al. translated the scale in Chinese with good reliability and validity tests [33]. It covers 3 domains, namely, vigor, absorption, and dedication, and 3 entries are set in every domain, with a total of 9 entries. A Likert 7-point scale was used, the score ranged from 0 (never) to 6 (always), with the overall score of 0–54, and a greater score indicated a better work engagement state. In the formal survey of this study, the scale had good internal consistency, with Cronbach's alpha coefficients of 0.855 for vigor, 0.957 for absorption, 0.901 for dedication, and 0.957 for overall work engagement.

#### 2.2.3. Career Calling

The Career Calling Scale proposed by Dobrow et al. in 2011 was utilized to measure career calling [34]. The applicability of the scale in the Chinese cultural context was confirmed by Pei et al. [35] and was revised by Shen Ming et al. [36], which was highly reliable and valid in the nurse population. The scale consisted of 12 entries. The 5-point Likert scale was used, with the score between 1 (strongly disagree) and 5 (strongly agree), and a greater score suggested a greater career calling level. The Cronbach's alpha coefficient of the scale was 0.971 in this study.

### 2.3. Statistical Analysis

SPSS 26.0 and AMOS 24.0 statistical software were employed for data analysis [37]. Descriptive statistics were first used to analyze the features of participants and measurement variables. Pearson's correlation coefficients were adopted for analyzing the relationships between organizational silence, work engagement, and career calling. The mediating role of career calling in the relationship between organizational silence and work engagement was examined using two-step structural equation modeling (SEM) [38]. In Step 1, the measurement model was tested to evaluate Hypothesis 1. In Step 2, the structural model was tested to assess Hypothesis 2. The overall model goodness of fit was assessed using the following criteria: a root mean square error of approximation (RMSEA) ≤ 0.06, a *χ*^2^/df ≤ 5.0, a Tucker–Lewis index (TLI) ≥ 0.95, a comparative fit index (CFI) ≥ 0.95, as well as a normed fit index (NFI) ≥ 0.90 [39, 40]. During model refinement, the modification index (MI) and estimated parameter changes were used to help select parameters that could be incorporated in the model to improve fit [41]. Based on the MIs, as well as the covariance between variables, the model was progressively revised by iteratively observing the MIs after each modification until adequate model fit was achieved [39, 40, 42]. A bias-corrected bootstrap test with 2000 random samples was conducted for testing direct/indirect effects [43].

## 3. Results

### 3.1. Participant Demographic Data

Altogether 1996 questionnaires were collected, among which, 32 were submitted within 2 min, 40 within more than 15 min, 38 had the same answers or the same pattern of answers for all items (such as repeated answers 1, 2, and 3), and 28 were submitted with missing items. Thus, 1858 questionnaires with complete data were eligible for data analysis, and the effective recovery rate (1858/1996) was 93.1%. Among the 1858 participants, the mean age was 34.25 (SD = 5.28) years.  Table 1 presents participant demographic information.

### 3.2. Descriptive Statistics and Variable Correlation Analysis

Table 2 shows the descriptive statistics and variable correlation analysis in the hypothesized model. The results showed that organizational silence had negative correlation with work engagement (*r* = −0.427, *p* < 0.01) and career calling (*r* = −0.469, *p* < 0.01), while career calling was positively correlated with work engagement (*r* = 0.792, *p* < 0.01).

### 3.3. Verification of Our Hypotheses

**Figure 1:**
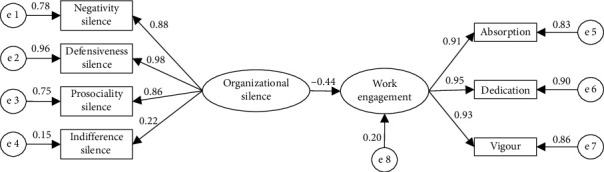
Direct effect model of organizational silence related to nurses' work engagement. Goodness-of-fit indices: RMSEA = 0.046, *χ*^2^/df = 4.9, TLI = 0.993, CFI = 0.995, and NFI = 0.994. The path coefficient is standardized estimates, *p* < 0.01.

First, the direct effect model was tested to examine whether organizational silence was negatively associated with nurses' work engagement (H1). The direct effect model showed a good fit to the data (RMSEA = 0.046, *χ*^2^/df = 4.9, TLI = 0.993, CFI = 0.995, and NFI = 0.994), with the path coefficients being statistically significant (*p* < 0.01). As shown in Figure 1, organizational silence was negatively associated with nurses' work engagement (*β* = −0.44, *p* < 0.01), explaining 19.6% of the variance in work engagement. A bootstrap analysis with 2000 samples revealed the 95% CI for this effect ranged from −0.3923 to −0.4908, further confirming statistical significance.

**Figure 2:**
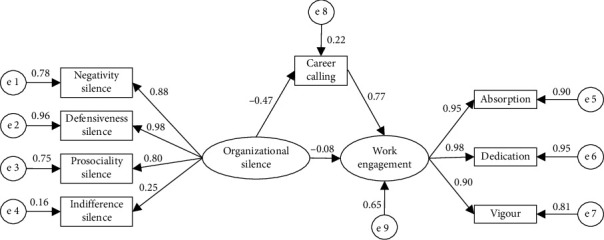
Mediation model with career calling as an mediator. Goodness-of-fit indices: RMSEA = 0.045, *χ*^2^/df = 4.78, NFI = 0.994, CFI = 0.995, and TLI = 0.992. All path coefficients are standardized estimates, *p*  < 0.01.

Subsequently, the mediation model was tested to examine whether career calling mediates the relationship between organizational silence and work engagement (H2). The initial mediation model had poor fit indices (RMSEA = 0.0618, *χ*^2^/df = 8.09, NFI = 0.989, CFI = 0.990, and TLI = 0.985), prompting refinement. This model was further improved by considering MIs and correlation among variables, that is, each modification was tested for correlation before iterative incorporation, and the model fit improved with each iteration [40]. Among the variables, e2 (error term of defensiveness silence) was correlated with e4 (error term of indifference silence) with MIS (13.984) and Par Change (−0.6175), and e5 (error term of absorption) was correlated with e6 (error term of dedication) with MIS (11.074) and Par Change (−0.106). The MIS considered in the study was from the same scale, so it was theoretically reasonable to have a high covariance. By correlating theoretically related error terms based on MIS, the final model improved substantially (RMSEA = 0.045, *χ*^2^/df = 4.78, NFI = 0.994, CFI = 0.995, TLI = 0.992), displayed in Figure 2.

In the final model, organizational silence was negatively related to career calling (*β* = −0.47, *p* < 0.01), thereby positively affecting work engagement (*β* = 0.77, *p* < 0.01). And after including career calling, organizational silence was still remarkably associated with work engagement (*β* = −0.08, *p* < 0.01), meaning that career calling played a partial mediating role.  Table 3 displays the estimates of the modified model parameters for nurses' work engagement. Organizational silence and career calling together explained 64.7% of the variance in work engagement, and organizational silence explained 21.8% of the variance in career calling.

Bootstrapping confirmed that the indirect effect of organizational silence on work engagement through career calling was statistically significant (indirect effect = 0.358; 95% CI did not contain zero), accounting for approximately 82% of the total effect, as shown in Table 4. These results provide strong evidence for the hypothesized mediating role of career calling.

## 4. Discussion

Organizational silence is characterized by employees' lack of interaction with the organization and unwillingness to make suggestions for the development of the organization [11]. This state not only reflects employees' psychological withdrawal, but is also closely related to their work behavior [9, 12]. In this study, the mediating path of career calling was proposed based on the JD-R model, and the mechanism of the role of organizational silence on clinical nurses' work engagement was explored.

In this study, organizational silence was found to be significantly negatively correlated with nurses' work engagement, validating Hypothesis 1. This result is consistent with existing studies [9, 15]. It indicated that the higher the organizational silence of nurses, the lower their level of work engagement. According to the JD-R model of work engagement, job resources are the core variables affecting work engagement [1], and good interpersonal relationships, effective task organization, and work autonomy are effective work resources for employees to increase their work engagement [1, 44], and this theoretical framework has been similarly validated in healthcare settings [45]. Organizational silence can lead to poor interpersonal relationships and impeded communication among employees, reducing their ability to take control of their own work and weakening employees' access to job resources, which in turn affects their autonomy, problem-solving ability, and task completion efficiency, ultimately leading to a decrease in the level of work engagement [12, 46]. Nursing is a high-risk, high-responsibility profession, and the destructive effects of organizational silence on nursing staff's job resources may be even more pronounced. For example, a culture of organizational silence may be result in nurses' reluctance to report errors, impede open lines of communication, and diminish nurses' participation and autonomy in scheduling and care decisions [47–49]. All of these factors may reduce nurses' access to work resources, thus decreasing their work engagement. A study on a group of Jordanian nurses also showed that adequate job resources, positive feedback in the healthcare environment enhanced nurses' work engagement [45].

Further analyses showed revealed a negative correlation between career calling and organizational silence, and a positive correlation between career calling and work engagement. These findings serve to partially mediate the relationship between organizational silence and work engagement, thereby verifying Hypothesis 2. This indicates that the lower the degree of organizational silence of the nurses, the higher the degree of career calling and the higher the degree of work engagement. Furthermore, it demonstrates that the degree of organizational silence among nurses can indirectly influence work engagement through career calling. These results are consistent with previous findings that organizational silence is negatively related to career calling [21] and that career calling is positively related to work engagement [25, 50].

Career calling is a highly intrinsic psychological resource, reflecting the degree to which an individual identifies with the significance of his or her work, social value, and self-fulfillment [19, 20]. As demonstrated in preceding studies, career calling has been shown to increase employees' career satisfaction, wellbeing, and engagement [51, 52] and to alleviate burnout [24]. To the best of our knowledge, this study was the first to explore the relationship between organizational silence, career calling, and work engagement among clinical nurses. The results indicated that career calling partially mediated the relationship between organizational silence and work engagement among clinical nurses. This finding is consistent with previous studies showing that career calling mediates the relationship between organizational environments and job performance [28, 29]. Organizational silence may reduce the ways in which employees can effectively deal with conflict or disputes, make it difficult for individuals to balance their beliefs and behaviors, and reduce employees' perceptions of the value of their work and their motivation to work, which in turn leads to lower levels of career calling and affects their work engagement [12, 46]. If nurses can offer ideas and suggestions for organizational development and express their concerns or worries about the organization, this may enhance the meaning and value of their work and increase their level of career calling, which in turn will stimulate their motivation and dedication to work and improve their work engagement. In a sample of Chinese nurses, nurses' career calling was positively related to work engagement [53]. A study in a Western country suggested that career calling may motivate nurses to be engaged in their work even in a demanding environment [54]. Thus, career calling partially mediates the relationship between organizational silence and work engagement, and nursing managers can increase clinical nurses' work engagement by reducing organizational silence and enhancing nurses' level of career calling.

In summary, organizational silence not only directly affects clinical nurses' work engagement but also indirectly affects the relationship between the two through career calling.

### 4.1. Implication for Nursing Management

This study offers associated implications for nursing management. For one thing, hospital managers must reduce organizational silence to promote nurses' work engagement. Managers should encourage employees to express their relevant ideas, information, and opinions; for example, by creating a supportive organizational climate and culture where employees feel safe to speak their minds, establishing effective communication channels among employees, encouraging employees' personal initiative by rewarding and incentivizing new ideas that help deal with organizational problems and help the organization grow, and promoting the exchange of ideas and information in the organization [12, 55]. For another, career calling is related to both organizational silence and work engagement, and it plays a mediating role in their relationship. Therefore, managers can adopt interventions aiming at increasing the level of nurses' career calling to improve their work engagement. For example, nursing managers create an environment in which nurses are respected and take measures to increase the perceived professional benefits among nurses, which promote their professional identity, job satisfaction, and well-being, thereby increasing the level of nurses' career calling [56, 57].

In addition, government administrators in China could strengthen public awareness of nurses' contributions to health, thereby improving societal recognition of their work and enhancing career calling [25, 58]. All these measures will promote nurses' work engagement and improve the quality of nursing care.

### 4.2. Limitations

Our study has several limitations. First, because of the cross-sectional nature, no causal interpretation was drawn. In future research, we should consider a longitudinal design to explore organizational silence in hospitals and the effect of organizational silence on nurses' career calling and work engagement. Second, the studied variables were reported by nurses themselves, which might lead to response bias. Therefore, more investigations must include multisource data for validating the relationship between organizational silence, career calling, and work engagement. Third, the results of this study were based on data collected in Chongqing, China. The generalizability of the findings was limited due to the socioeconomic and cultural characteristics of the regions. Therefore, future studies should collect data in multiple regions to improve the generalizability of our findings.

## 5. Conclusions

Chinese nurses' organizational silence and career calling are not only related to work engagement but also have significant predictive effects on work engagement. Furthermore, career calling exerts a mediating effect on the relation of organization silence with work engagement. Therefore, hospital managers in China should focus on reducing organizational silence and fostering career calling as effective strategies to enhance nurses' work engagement.

## Figures and Tables

**Table 1:** Participant demographic information (*n* = 1858).

Variables	*n* (%)
Gender	
Male	101 (5.4%)
Female	1757 (94.6%)
Education level	
Junior college and less	159 (8.6%)
Undergraduate	1616 (87.0%)
Postgraduate and more	83 (4.4%)
Marital status	
Single	467 (25.1%)
Married	1330 (71.6%)
Divorced	61 (3.3%)
Age	
< 30 years	478 (25.7%)
30–40 years	1142 (61.5%)
41–50 years	184 (9.9%)
> 50 years	54 (2.9%)
Working years	
< 3 years	481 (25.9%)
3–5 years	439 (23.6%)
> 5 years	938 (50.5%)

**Table 2:** Mean scores and bivariate correlations between variables (*n* = 1858).

	Mean (SD)	Range	1	2	3	4	5	6	7	8	9	Cronbach's a
(1) Negativity silence	11.48 (5.538)	6–30	—									0.937
(2) Defensiveness silence	11.39 (5.959)	6–30	0.866^∗∗^	—								0.959
(3) Prosociality silence	8.07 (4.193)	4–20	0.755^∗∗^	0.850^∗∗^	—							0.941
(4) Indifference silence	6.25 (3.058)	4–20	0.222^∗∗^	0.208^∗∗^	0.211^∗∗^	—						0.900
(5) Organizational silence	37.18 (15.757)	20–100	0.923^∗∗^	0.949^∗∗^	0.894^∗∗^	0.407^∗∗^	—					0.914
(6) Vigor	10.88 (2.850)	0–18	−0.395^∗∗^	−0.391^∗∗^	−0.327^∗∗^	−0.110^∗∗^	−0.395^∗∗^	—				0.855
(7) Dedication	11.62 (2.756)	0–18	−0.418^∗∗^	−0.411^∗∗^	−0.339^∗∗^	−0.139^∗∗^	−0.420^∗∗^	0.881^∗∗^	—			0.901
(8) Absorption	10.80 (3.013)	0–18	−0.411^∗∗^	−0.398^∗∗^	−0.344^∗∗^	−0.113^∗∗^	−0.408^∗∗^	0.846^∗∗^	0.865^∗∗^	—		0.933
(9) Work engagement	33.31 (8.218)	0–54	−0.428^∗∗^	−0.419^∗∗^	−0.353^∗∗^	−0.127^∗∗^	−0.427^∗∗^	0.953^∗∗^	0.958^∗∗^	0.950^∗∗^	—	0.957
(10) Career calling	46.96 (10.260)	12–60	−0.468^∗∗^	−0.451^∗∗^	−0.387^∗∗^	−0.160^∗∗^	−0.469^∗∗^	0.720^∗∗^	0.773^∗∗^	0.773^∗∗^	0.792^∗∗^	0.971

^∗^ *p* < 0.05.

^∗∗^ *p* < 0.01.

**Table 3:** Parameter estimates of this modified model.

Dependent variable	Independent variable	Parameter estimate	Standard error	Critical ratio	Standardized estimate	Squared multiple correlation
Work engagement	Organizational silence	−0.044	0.009	−4.722	−0.076	0.647
	Career calling	−0.2133	0.005	45.532	0.766	

Career calling	Organizational silence	−0.982	0.045	−21.616	−0.467	0.218

**Table 4:** Standardized direct, indirect, and total effects of our modified model.

Dependent variable	Independent variable	Direct effect (95% CI)	Indirect effect (95% CI)	Total effect (95% CI)
Work engagement	Organizational silence	−0.076 (−0.044∼−0.109)	−0.358 (−0.324∼−0.395)	−0.434 (−0.385∼−0.482)
	Career calling	0.766 (0.732∼0.797)	—	0.766 (0.732∼0.797)

Abbreviation: CI, Confidence interval.

## Data Availability

The data that support the findings of this study are available from the corresponding author upon reasonable request.
